# Electrochemical DNA Sensor for Sensitive BRCA1 Detection Based on DNA Tetrahedral-Structured Probe and Poly-Adenine Mediated Gold Nanoparticles

**DOI:** 10.3390/bios10070078

**Published:** 2020-07-20

**Authors:** Dezhi Feng, Jing Su, Guifang He, Yi Xu, Chenguang Wang, Mengmeng Zheng, Qiuling Qian, Xianqiang Mi

**Affiliations:** 1 Shanghai Advanced Research Institute, Chinese Academy of Sciences, Shanghai 201210, China; fengdezhi@sari.ac.cn (D.F.); heguifang@sari.ac.cn (G.H.); xuyi@sari.ac.cn (Y.X.); wangcg@sari.ac.cn (C.W.); zhengmengmeng2018@sari.ac.cn (M.Z.); qianqiuling2018@sari.ac.cn (Q.Q.); 2 University of Chinese Academy of Sciences, Beijing 100049, China; 3 Institute for Personalized Medicine, School of Biomedical Engineering, Shanghai Jiao Tong University, Shanghai 200030, China; sujing@sjtu.edu.cn; 4 School of Life Sciences, Shanghai University, Shanghai 200444, China; 5 Key Laboratory of Functional Materials for Informatics, Shanghai Institute of Microsystem and Information Technology, Chinese Academy of Sciences, Shanghai 200050, China; 6 CAS Center for Excellence in Superconducting Electronics (CENSE), Shanghai 200050, China; 7 Key Laboratory of Systems Biology, Hangzhou Institute for Advanced Study, University of Chinese Academy of Sciences, Chinese Academy of Sciences, Hangzhou 310024, China

**Keywords:** electrochemical DNA sensor, DNA tetrahedral structured probe, sandwich system, BRCA1, polyA-gold nanoparticles

## Abstract

BRCA1 is the biomarker for the early diagnosis of breast cancer. Detection of BRCA1 has great significance for the genetic analysis, early diagnosis and clinical treatment of breast cancer. In this work, we developed a simple electrochemical DNA sensor based on a DNA tetrahedral-structured probe (TSP) and poly-adenine (polyA) mediated gold nanoparticles (AuNPs) for the sensitive detection of BRCA1. A thiol-modified TSP was used as the scaffold on the surface of the screen-printed AuNPs electrode. The capture DNA (TSP) and reporter DNA were hybridized to the target DNA (BRCA1), respectively, to form the typical sandwich system. The nanocomposites of reporter DNA (polyA at the 5′ end) combined with AuNPs were employed for signal amplification which can capture multiple enzymes by the specificity between biotin and streptavidin. Measurements were completed in the electrochemical workstation by cyclic voltammetry and amperometry and we obtained the low limit of detection of 0.1 fM with the linear range from 1 fM to 1 nM. High sensitivity and good specificity of the proposed electrochemical DNA sensor showed potential applications in clinical early diagnosis for breast cancer.

## 1. Introduction

Breast cancer is one of the most common malignancies and the major leading cause of cancer death among females in over 100 countries [1]. It is indispensable to diagnose breast cancer in the early stage. The breast cancer susceptibility gene (BRCA1) is a human tumor-suppressor gene that is involved in DNA damage repair [2]. Researchers have identified that mutations in BRCA1 are associated with a high risk of inherited breast cancer for 40–50% of cases [3,4]. Thus, the detection of BRCA1 is of great significance for the genetic analysis, early diagnosis and clinical treatment of breast cancer. The traditional detection methods of BRCA1 include single-strand conformation polymorphism assay (SSCP), high-performance liquid chromatography (HPLC) and DNA sequencing [5,6,7,8]. However, these methods have limitations in analytical time, cost and simplicity. It is necessary to develop a rapid, sensitive, low-cost and simple method for BRCA1 detection.

In recent years, the development of electrochemical biosensors for DNA biomarkers has been of increasing interest to researchers due to their fast-analytical time, low cost and ease of miniaturization compared with traditional detection methods [9,10,11,12,13,14]. However, as the capture probe for electrochemical DNA sensor, single-stranded DNA is not rigid and lacks the ability to modulate the density of probes. In order to improve the detection sensitivity, the DNA tetrahedral-structured probe (TSP) has been widely used to develop electrochemical DNA sensors with well-controlled density and orientation, which result from its rigid and three-dimensional structure [15,16]. Fan’s group reported various electrochemical DNA sensors based on DNA tetrahedral nanostructures for the sensitive detection of biomarkers of nucleic acids and proteins [17,18,19]. Zeng developed a new electrochemical DNA sensor based on double DNA tetrahedral nanostructures with sandwich systems for the sensitive detection of target DNA as low as 1 fM [20].

The application of gold nanoparticles (AuNPs) attracted more attention in the development of electrochemical DNA sensors because of their advantages, such as fast electron conduction, ease of labeling and good biocompatibility [21,22,23,24]. Rasheed developed an electrochemical DNA sensor for BRCA1 detection based on a sandwich system and AuNPs to achieve signal amplification [25]. In another study by Rasheed, BRCA1 was detected using hybridization among functionalized AuNPs-labeled probes and the bond of DNA to AuNPs which benefited from the hydrogen bonding [26]. Compared with traditional protocols for bonding DNA and AuNPs together, poly-adenine (polyA) can provide the higher orientation controllability and hybridization efficiency of the probe [27,28]. According to our previous report, AuNPs and polyA were constructed together to mediate the nanoscale molecular beacon (MB) for detection of multiple miRNAs [29]. Liu proposed an electrochemical DNA sensor for adenosine rapid detection through a polyA-mediated rolling motor and TSPs [30].

In this work, we developed a simple electrochemical DNA sensor based on TSP and polyA mediated AuNPs for the sensitive detection of BRCA1. Firstly, a screen-printed electrode was used as the substrate of the sensor and the electrode surface was electrodeposited with AuNPs for larger specific surface area and faster signal response. Secondly, the capture DNA (DNA-c, an extended DNA of the top of TSP) and reporter DNA (DNA-r) were hybridized to half of the target DNA (DNA-t, BRCA1), respectively, to form the stable sandwich system, and so DNA-r (polyA at the 5′ end) was labeled with AuNPs to achieve signal amplification. Finally, horseradish peroxidase modified with streptavidin (SA-HRP) was linked to DNA-r which was modified with biotin at the 3′ end. The electrochemical detection of BRCA1 was completed by cyclic voltammetry (CV) and amperometry (IT).

## 2. Materials and Methods

### 2.1. Materials and Instruments

The DNA oligonucleotides were synthesized by Sangon (Shanghai, China). The sequences are shown in Table S1 in supplementary materials. 3,3′,5,5′-tetramethylbenzidine with hydrogen peroxide (TMB, H_2_O_2_ included) and SA-HRP were purchased from Sigma-Aldrich (St. Louis, Mo, USA). Bovine serum albumin (BSA), casein, tween-20, tris (2-carboxyethyl) phosphine hydrochloride (TCEP), KCl, NaCl, Na_2_HPO_4_, KH_2_PO_4_ and other chemical reagents were all purchased from Sinopharm Chemical Reagent Co. Ltd. (Shanghai, China). HAuCl_4_ and AuNPs solution were purchased from Bailingwei Technology Co., Ltd. (Shanghai, China) and Yuanmai Biological Technology Co., Ltd. (Shanghai, China). PB solution (10 mM, 200 mM, pH = 7.6), PBS solution (100 mM, pH = 7.6), PBST solution (PBS, tween-20 included), TE buffer (10 mM Tris, 1 mM EDTA, pH 8.0) and TM buffer (20 mM Tris, 50 mM MgCl_2_, pH 8.0) were all prepared with Milli-Q water (18.2 MΩ·cm resistivity). 16-channel screen-printed electrodes (16-SPE) and the HSBS16x electrochemical workstation were purchased from HuasenXinke (Suzhou) Nanotechnology Co. Ltd. (Suzhou, China) Microscope images were obtained by a Multimode-8 atomic force microscope (AFM), a Nova Nano 450 scanning electron microscope (SEM) and a JEM-1400Plus transmission electron microscope (TEM). Absorption spectra were recorded by an UH5300 ultraviolet-visible (UV-vis) spectrophotometer.

### 2.2. Synthesis of Tetrahedral-Structured Probes (TSPs)

The synthesis of TSPs was according to reported protocols [31]. Four single-stranded DNA oligonucleotides were dispersed in TE buffer, forming a final concentration of 100 μM. 1 μL of each strand was combined with 10 μL TCEP (30 mM) and 86 μL 1 X TM buffer to be heated at 95 °C for 10 min, then at 4 °C for 30 s. The final concentration of TSPs was 1 μM.

### 2.3. Synthesis the Nanocomposites of Gold Nanoparticles (AuNPs)-DNA-r

AuNPs-DNA-r composites were synthesized according to the previous reports [29]. The temperature during the synthesis was at room temperature. First, the DNA reporter probes were added to the AuNPs solution (15 nm) for 10 min. Second, citrate·HCl buffer (500 mM, pH = 3) was added to the mixed solution to reach the final concentration of 10 mM for 5 min. Then, PB solution (200 mM) was added to the mixed solution for 15 min. Finally, PB solution (10 mM) was used to wash the mixed solution after centrifugation at 13,000 rpm for three times to remove the redundant unassembled probes. The nanocomposites of AuNPs-DNA-r were obtained after re-dispersion in PBS solution to reach the final concentration of 2 nM.

### 2.4. Development of Electrochemical DNA Sensor

Prior to modification, 16-SPE was cleaned with 1X PBS and dried under N_2_. A layer of AuNPs at the surface of electrode was obtained by electrodeposition at −100 mV and 100 mV/s in HAuCl_4_ solution for 300 s. The electrode was placed into 1 μM TSPs solution for 12 h at room temperature to form SPE/TSPs electrode. Then, each electrode was dipped in blocking buffer (1% casein and BSA in PBS) at 37 °C for 2 h. Subsequently, the SPE/TSPs electrode was dipped in the nanocomposites of BRCA1/AuNPs-DNA-r at 37 °C for 2 h. After being cleaned with PBS and dried under N_2_, the electrode was dipped in SA-HRP at 37 °C for 1 h to form the SPE/TSPs/BRCA1/AuNPs-DNA-r/SA-HRP electrode. The electrode was then cleaned with 1X PBST solution three times for three minutes each time. Finally, the electrode was placed into the electrochemical workstation for CV and IT measurements. The CV parameters were as follows: initial E, 0.7 V; high E, 0.7 V; Low E, 0 V; initial scan, negative; scan rate, 0.1 V/s; sweep segments, 2; sample interval, 0.001 V; quiet time, 2 s; sensitivity, 2 × 10^−5^ A/V. The parameters for ITs were as follows: initial E, 0.1 V; sample interval, 0.1 s; run time, 200 s; quiet time, 0 s; sensitivity, 2 × 10^−5^ A/V.

## 3. Results and Discussion

### 3.1. Principle of the Electrochemical DNA Sensor

**Figure 1:**
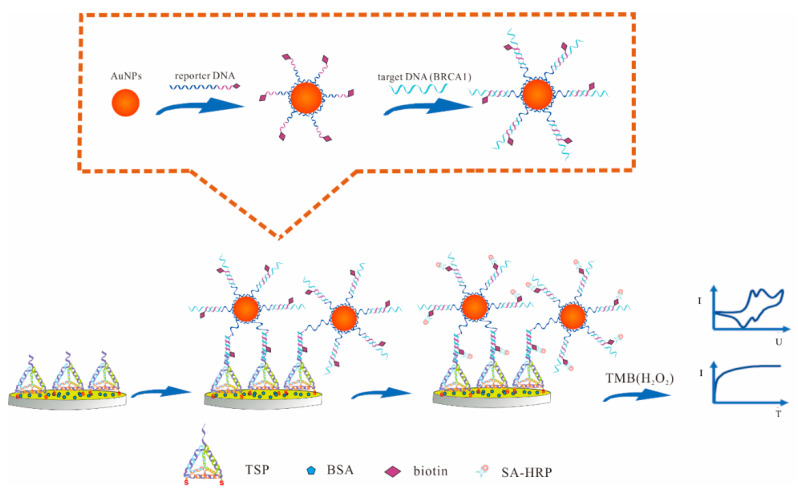
The principle of the development of electrochemical DNA sensor. Cyclic voltammetry (CV curve) and amperometry (IT curve) were applied to investigate the electrochemical behavior of the proposed electrochemical DNA sensor.

The principle of the proposed electrochemical DNA sensor for BRCA1 detection was based on the electrochemical signals of the redox reaction in the presence of the TMB. As shown in Figure 1, firstly, TSP was immobilized on the surface of the AuNPs electrode through the Au–S bond due to the thiol groups being modified at the three of its vertices. Secondly, the typical sandwich system was formed by the hybridization of DNA-c, DNA-t and DNA-r, which helped to effectively immobilize the DNAs. Then, we assembled the AuNPs and DNA-r (polyA at the 5′ end) together based on the adsorption between adenine and Au, and one AuNP could adsorb multiple strands of DNA-r to achieve signal amplification. With the affinity between biotin and SA, the biotin modified DNA-r could bind to SA-HRP specifically, which catalyzes the reduction of hydrogen peroxide and generates quantitative electrochemical current signals in the presence of the co-substrate, 3, 3′, 5, 5′ tetramethylbenzidine (TMB) [19]. Thus, the proposed electrochemical detection of BRCA1 could be easily achieved.

### 3.2. Characterization of TSPs and AuNPs Electrode

**Figure 2:**
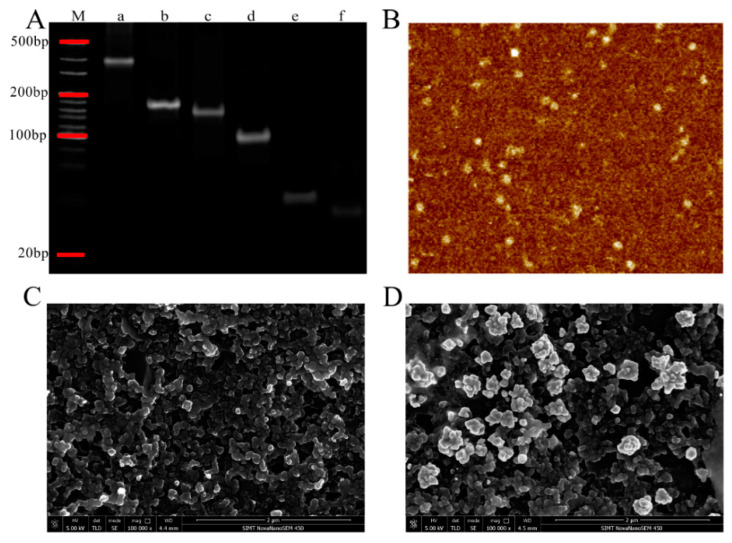
(**A**) Gel electrophoresis image of marker (lane M), tetrahedral-structured probes (TSPs, lane a), ABC (lane b), BCD (lane c), AB (lane d), A (lane e), and B (lane f). (**B**) Atomic force microscopy (AFM) image of TSPs. Scanning electron microscopy (SEM) results of (**C**) bare carbon electrode and (**D**) gold nanoparticles (AuNPs) electrode. The scale value was 2 μm.

Polyacrylamide gel electrophoresis (PAGE) and atomic force microscopy (AFM) were employed to characterize the formation of TSPs. As shown in Figure 2A, the molecular weight of six lanes was in descending order from lane a to lane f, which represented the TSP, triple-stranded DNA (ABC, BCD), double-stranded DNA (AB) and single-stranded DNA (A, B) respectively. The electrophoresis rate of A (66 bases) was lower than B (55 bases, equaled to C and D) due to the difference in molecular weight, which caused the TSP with complex structure to shift slower than the structures of triple-stranded DNA and double-stranded DNA. Figure 2B shows the AFM image of TSPs. The shape of triangle indicated the tetrahedral structure of TSPs. All these results showed the successful formation of the TSPs.

Scanning electron microscopy (SEM) was performed to characterize the surface of SPEs. As shown in Figure 2C, the surface of the bare carbon electrode was relative smooth and it was easy of modification. The highlighted particles indicated the successful deposition of AuNPs and the rough surface compared with bare carbon electrode is showed in Figure 2D, which increased the specific surface area. AuNPs on the surface of the electrode could enhance the amount of DNA that fixed on the electrode surface.

### 3.3. Characterization of the AuNPs-DNA-r

**Figure 3:**
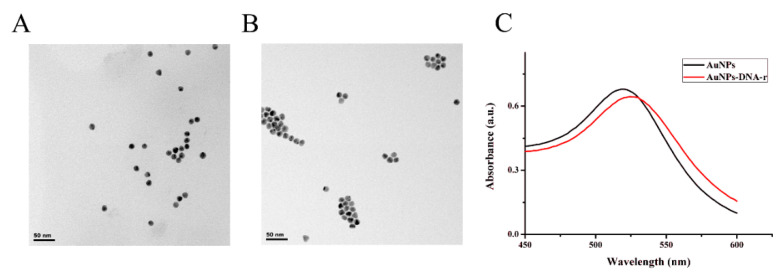
Transmission electron microscope (TEM) images of (**A**) AuNPs and (**B**) AuNPs-DNA-r. The scale value was 50 nm. (**C**) The ultraviolet-visible (UV-vis) absorption spectra of the AuNPs and the AuNPs-DNA-r. Black curve indicates the AuNPs and red curve indicates the AuNPs-DNA-r.

Transmission electron microscope (TEM) was performed to characterize the nanocomposites of AuNPs-DNA-r. As shown in Figure 3A, the naked AuNPs were in a state of dispersion. Although the state of dispersion of the nanocomposites of the AuNPs-DNA-r was not obvious in Figure 3B, these particles were actually dispersed, which indicated that the nanocomposites of AuNPs-DNA-r were successfully assembled. We could find that the nanocomposites had a better dispersibility and uniform distribution after sonication before sample preparation with different scale value of 50 nm and 100 nm in Figure S1. From UV-vis absorption spectra in Figure 3C, we found that the maximum absorption peak of the AuNPs shifted slightly from 520 nm to 525 nm, which also confirmed that the successful assembly of the AuNPs-DNA-r.

### 3.4. Optimization of the Experimental Conditions

**Figure 4:**
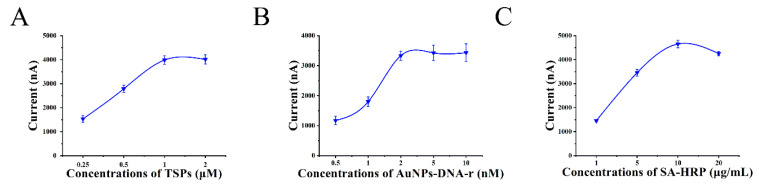
The optimization of the concentration of (**A**) TSPs, (**B**) AuNPs-DNA-r, (**C**) streptavidin (SA-HRP). Error bars show the standard deviations (*n* = 4).

Some influencing factors such as the concentrations of TSPs, AuNPs-DNA-r, and SA-HRP were studied. The introduction of TSPs provided the scaffold of the sandwich system and enhanced the detection sensitivity. As shown in Figure 4A, the current increased along with the increase of the concentrations of TSPs and reached to a maximum at 1 μM which was higher than the current of the concentration of 2 μM. Then, 1 μM was selected as the optimal concentration. The nanocomposites of AuNPs-DNA-r had an important effect on signal amplification and benefited the formation and stability of the sandwich system. At the optimal concentration of TSPs, the current increased significantly when the AuNPs-DNA-r concentration increased while the current increased slightly when the concentration was higher than 2 nM, suggesting that the optimal concentration of AuNPs-DNA-r was 2 nM (Figure 4B). Furthermore, the concentration of SA-HRP produced signal catalysis and the sensitivity. As show in Figure 4C, the current increased to a maximum at the concentration of 10 μg/mL, and then decreased, suggesting that 10 μg/mL was the optimal concentration.

### 3.5. Performance of the Electrochemical DNA Sensor

**Figure 5:**
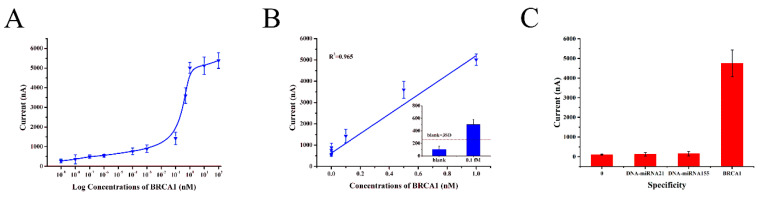
(**A**) The plots of currents versus the target DNA (BRCA1) concentrations: 0, 1 aM, 10 aM, 100 aM, 1 fM, 100 fM, 1 pM, 100 pM, 500 pM, 1 nM, 10 nM, 100 nM. (**B**) The linear calibration curve for BRCA1 detection with the concentrations of 1 fM, 100 fM, 1 pM, 100 pM, 500 pM and 1 nM. Inset: Histogram showing the limit of detection of BRCA1 detection by the electrochemical DNA sensor, and the dashed lines stand for the threshold (blank + 3SD). (**C**) Specificity of the proposed electrochemical DNA sensor. The concentrations of DNA-miRNA21 and DNA-miRNA155 are 1 μM while the BRCA1 is 1 nM. Error bars show the standard deviations (*n* = 4).

Under optimal experimental conditions, the proposed electrochemical DNA sensor for BRCA1 detection was examined by CV and IT, and the current was obtained in the presence of TMB. Figure 5A showed the relationship between the current and target. The current increased gradually with the BRCA1 concentration range of 0–100 nM while it increased slightly when the concentration was higher than 1 nM. In Figure 5B, the results showed that the linear range was from 1 fM to 1 nM with the equation of the calibration curve: Y = 4573.27X + 626.66, R^2^ = 0.965 and the limit of detection was 0.1 fM whose signal was higher than the blank signal plus three standard deviations (3SD) [32] as shown in Figure 5B inset. The high sensitivity may be attributed to the introduction of TSPs and the nanocomposites of AuNPs-DNA-r, which was higher than the sensitivity of recent reports [33,34,35].

To investigate the specificity of the proposed electrochemical DNA sensor, control experiments were performed using blank, DNA-miRNA21 and DNA-miRNA155 (Figure 5C). It was noticed that the low concentration of BRCA1 which matched perfectly generated prominent signals while DNA-miRNA21 and DNA-miRNA155 exhibited weak signal approaching the level of the zero-concentration control. The excellent specificity maybe attributed to the stable sandwich system in this sensor.

## 4. Conclusions

We have reported a simple electrochemical DNA sensor for the sensitive detection of BRCA1, which achieved the linear range of 1 fM to 1 nM and the detection limit of 0.1 fM. PAGE, AFM and TEM measurements indicated the successful formation of TSPs and the successful assembly of AuNPs-DNA-r. The sandwich system assured the stability of the DNAs on the surface of the sensor. The detection signal of the sensor was effectively enhanced through the combination of AuNPs and DNA-r. This sensor showed good specificity against blank and non-complementary sequences (DNA-miRNA21, 155). We believe the proposed electrochemical DNA sensor could prove its potential application in the early diagnosis of cancer.

## Supplementary Materials

The following are available online at https://www.mdpi.com/2079-6374/10/7/78/s1: Figure S1: Characterization of the AuNPs-DNA-r; Table S1. Sequences used in the work.
