# Supplementary material for: Electrochemical DNA Sensor for Sensitive BRCA1 Detection Based on DNA Tetrahedral-Structured Probe and Poly-Adenine Mediated Gold Nanoparticles
Source: Biosensors (Basel). 2020 Jul 20;10(7):78. doi: 10.3390/bios10070078 (PMC7400266; doi:10.3390/bios10070078)
Supplement: Supplementary file 1 [file biosensors-10-00078-s001.pdf]

biosensors

MDPI

Supplementary Materials: Electrochemical DNA Sensor for Sensitive brca1 Detection Based on DNA Tetrahedral-Structured Probe and Poly-Adenine Mediated Gold Nanoparticles

Dezhi Feng [1,2,†], Jing Su [3,†], Guifang He [1,4], Yi Xu [1], Chenguang Wang [1,2], Mengmeng Zheng [1,4], Qiuling Qian [1,2] and XianQiang Mi [1,2,5,6,7,*]

1 Shanghai Advanced Research Institute, Chinese Academy of Sciences, Shanghai 201210, China; fengdezhi@sari.ac.cn (D.F.); heguifang@sari.ac.cn (G.H); xuyi@sari.ac.cn (Y.X.); wangcg@sari.ac.cn (C.W.); zhengmengmeng2018@sari.ac.cn (M.Z.); qianqiuling2018@sari.ac.cn (Q.Q.)
2 University of Chinese Academy of Sciences, Beijing 100049, China
3 Institute for Personalized Medicine, School of Biomedical Engineering, Shanghai Jiao Tong University, Shanghai 200030, China; sujing@sjtu.edu.cn
4 School of Life Sciences, Shanghai University, Shanghai, 200444, China
5 Key Laboratory of Functional Materials for Informatics, Shanghai Institute of Microsystem and Information Technology, Chinese Academy of Sciences, Shanghai 200050, China
6 CAS Center for Excellence in Superconducting Electronics (CENSE), Shanghai 200050, China
7 Key Laboratory of Systems Biology, Hangzhou Institute for Advanced Study, University of Chinese Academy of Sciences, Chinese Academy of Sciences, Hangzhou, 310024, China
* Correspondence: mixq@mail.sim.ac.cn
† D.F. and J.S. contributed equally to this work.

Table S1. Sequences used in the work.

	**Sequences (from 5' to 3')**
**DNA-t (BRCA1)**	GAACAAAAGGAAGAAAATC
**DNA-r**	AAAAAAAAAAAAAAAAAAAAAAAAAAAAAAGATTTTCTTC- biotin
**TSP-A**	CTTTTGTTCAAACATTCCTAAGTCTGAAACATTACAGCTTGCTACA CGAGAAGAGCCGCCATAGTA
**TSP-B**	SH-TATCACCAGGCAGTTGACAGTGTAGCAAGCTGTAATAGATGCGA GGGTCCAATAC
**TSP-C**	SH-TCAACTGCCTGGTGATAAAACGACACTACGTGGGAATCTACTA TGGCGGCTCTTC
**TSP-D**	SH-TTCAGACTTAGGAATGTGCTTCCCACGTAGTGTCGTTTGTATTG GACCCTCGCAT
**DNA-miRNA21**	TAGCTTATCAGACTGATGTTGA
**DNA-miRNA155**	TTAATGCTAATCGTGATAGGGG

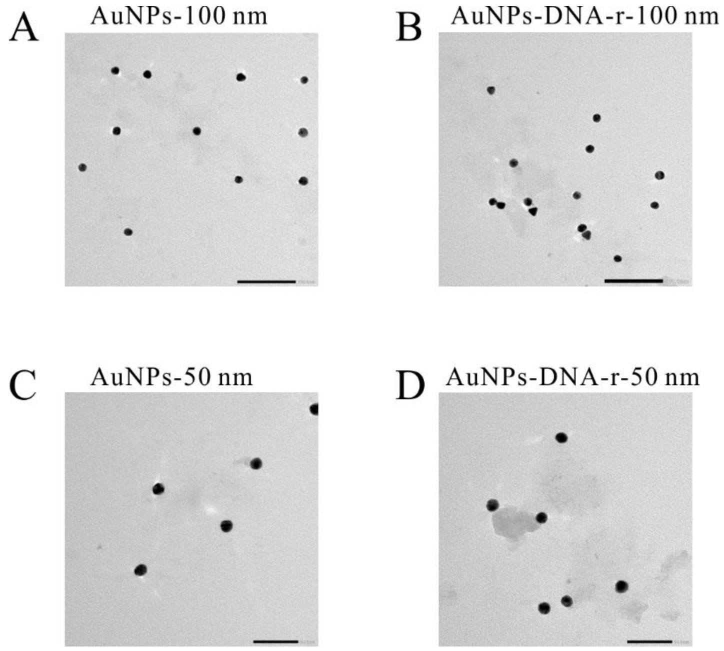

Figure S1. Characterization of the AuNPs-DNA-r. TEM images of (A) AuNPs and (B) AuNPs-DNA-r after sonication before sample preparation with the scale value of 100 nm. TEM images of (C) AuNPs and (D) AuNPs-DNA-r after sonication before sample preparation with the scale value of 50 nm.
